# Comparison of UHPLC and HPLC in Benzodiazepines Analysis of Postmortem Samples: A Case–Control Study

**DOI:** 10.1097/MD.0000000000000640

**Published:** 2015-04-10

**Authors:** Behnam Behnoush, Ardeshir Sheikhazadi, Elham Bazmi, Akbar Fattahi, Elham Sheikhazadi, Seyed Hossein Saberi Anary

**Affiliations:** From the Department of Forensic Medicine, School of Medicine, Tehran University of Medical Sciences (BB, AS); Tehran University of Medical Sciences, Faculty of Pharmacy, Toxicology and Poisoning Research Centre (AS); Laboratory of Forensic Toxicology, Legal Medicine Research Center of Tehran, Tehran, Iran (EB, AF); School of Nursing & Midwifery, Kermanshah University of Medical Sciences, Kermanshah, Iran (ES); Health Services Management, Kerman University of Medical Sciences, Kerman, Iran (SHSA).

## Abstract

The aim of this study was to compare system efficiency and analysis duration regarding the solvent consumption and system maintenance in high-pressure liquid chromatography (HPLC) and ultra high-pressure liquid chromatography (UHPLC).

In a case–control study, standard solutions of 7 benzodiazepines (BZs) and 73 biological samples such as urine, tissue, stomach content, and bile that screened positive for BZs were analyzed by HPLC and UHPLC in laboratory of forensic toxicology during 2012 to 2013. HPLC analysis was performed using a Knauer by 100-5 C-18 column (250 mm × 4.6 mm) and Knauer photodiode array detector (PAD). UHPLC analysis was performed using Knauer PAD detector with cooling autosampler and Eurospher II 100-3 C-18 column (100 mm × 3 mm) and also 2 pumps. The mean retention time, standard deviation, flow rate, and repeatability of analytical results were compared by using 2 methods.

Routine runtimes in HPLC and UHPLC took 40 and 15 minutes, respectively. Changes in mobile phase composition of the 2 methods were not required. Flow rate and solvent consumption in UHPLC decreased. Diazepam and flurazepam were detected more frequently in biological samples.

In UHPLC, small particle size and short length of column cause effective separation of BZs in a very short time. Reduced flow rate, solvent consumption, and injection volume cause more efficiency and less analysis costs. Thus, in the detection of BZs, UHPLC is an accurate, sensitive, and fast method with less cost of analysis.

## INTRODUCTION

Benzodiazepines (BZs) are the most frequently prescribed drugs and many of them are used for addiction, abuse, and sexual assault.^1,2^ Thus, their analysis seems to be more important in both clinical and forensic toxicology.^3^ Analysis of BZs with gas chromatography (GC) and gas chromatography–mass spectrometry (GC/MS) instrumentation is difficult, as they have nonvolatile and polar structures. Therefore, high-pressure liquid chromatography (HPLC) is ideally suited for their detection.^4^ Efficiency, speed, increased throughput, and reduced analysis cost are the important characteristics of HPLC. The goal of HPLC is to separate molecules in minimum time.^5,6^ Common methods available to shorten the analytical runs are shortening the column length, increasing the flow velocity, decreasing the particle size, and increasing the temperature. An ultra high-performance liquid chromatography (UHPLC) system is specially designed for higher pressure during chromatographic analysis with short column and small particle size. The aim of this study was to compare HPLC and UHPLC in the detection of BZs in biological and non-biological samples.

**FIGURE 1:**
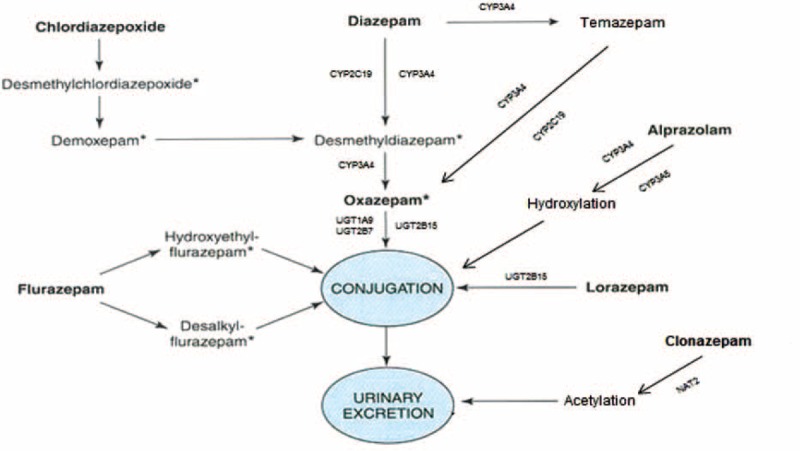
Benzodiazepine metabolites pathway.

BZs are mainly metabolized in 2 principle pathways: hepatic microsomal oxidation, *N*-dealkylation or aliphatic hydroxylation (phase Ι) and glucuronide conjugation (phase ΙΙ). CYP3A4_,_ CYP3A5, and CYP2C19 are important enzymes of biotransformation of BZs.^7^Figure 1 shows the biotransformation of BZs.

## MATERIALS AND METHODS

### Design and Setting

This study was conducted using standard solutions of 7 BZs and 73 biological samples that screened positive for BZs analyzed by using HPLC and UHPLC in the laboratory of forensic toxicology of Legal Medicine Organization (LMO) of Iran (2012–2013). The probability of postmortem redistribution in this study was not significant as the samples from liver tissue, stomach content, bile, and urine were taken within 12 hours after death.

### Ethical Review

According to the Tehran University of Medical Sciences Ethics Committee, written informed consent forms were completed by the patients or close relatives of the deceased, and they were assured that all the information about the bodies were fully confidential. The ethical principles of the Helsinki Declaration were followed by the authors.

### Chemicals and Reagents

HCl, NH_3_, chloroform, and isopropanol for liquid–liquid extraction were obtained from Merck (Gernsheim, Germany). Water, acetonitrile, and methanol were of HPLC grade and purchased from Merck. KH_2_PO_4_ and phosphoric acid were of analytical grade for the preparation of phosphoric buffer (pH = 2.32). Drug standards were obtained from APO (Toronto, Canada) and Roch (Basel, Switzerland).

### Instrumentation

HPLC analysis was performed using a Knauer equipped with a photodiode array detector (PAD) (S 2800, 4 channels). Separation of analytes was performed by using Eurospher 100-5 C-18 column (250 mm × 4.6 mm) (Knauer, Berlin, Germany) with an S-1000 pump. Gradient mode was low-pressure gradient and maximum flow and pressure were 10 ml/min and 400 bar, respectively. UHPLC analysis was achieved by using a Knauer PAD detector with cooling autosampler (PDA-1, 6 channels). Column was Eurospher II 100-3 C-18 (100 mm × 3 mm). There were 2 pumps: the first pump with degasser module and the second with mixing chamber. Gradient mode in both pumps was high-pressure gradient with 10 ml/min maximum flow and 750 bar maximum pressure. Autosampler was AS-1 (loop volume 10 μl, tubing volume 15 μl, and syringe volume 250 μl) and tray configuration in left and right was 48 vials with tray cooling.

The chromatographic software that we used was Ezchrom Elite in HPLC and UHPLC, chromatographic conditions for all above-mentioned stationary phases were applied by using the same mobile phase consisting of buffer phosphate (pH = 2.32) and acetonitrile (63:37). Different flow rates were applied and optimized for column so as to obtain the results as fast as possible. Analytical results were achieved by using SPSS Version 17.0. Chicago: SPSS Inc.

### Sample Preparation

Standard solutions of 7 BZs (chlordiazepoxide, flurazepam, oxazepam, lorazepam, clonazepam, alprazolam, and diazepam) were prepared by dissolving pure standard amounts in methanol (10 mg in 100 ml) and filtering by pore filters. All the biological samples that consisted of BZs (detected by using MN kit) were extracted with liquid–liquid extraction. Urine, tissue, stomach content, and bile were acidic with concentrated HCl (%37) until pH = 3. Then, samples were heated in a boiling water-bath (95°C) for 20 minutes and stored at room temperature for 24 hours. After adding concentrated NH_3_ to adjust the pH 9 to 12, extraction was carried out with chloroform–isopropanol (80:20) and the samples were dried. Samples were reconstituted with 100 μl methanol before injection.

The mean values of retention times (RTs) and standard deviation were determined in standard solutions of 7 BZs and biological samples (liver, stomach content, urine, etc.) by using HPLC and UHPLC. The chromatographic conditions originally developed for BZs are described in previous studies.^8^

The repeatability of analysis was tested on RT. The RT of an individual compound serves as a key factor for compound identification and is the most important parameter for the analysis time. The standard sample solutions were injected 3 times into chromatographic system in either HPLC or UHPLC under optimized conditions. The linearity of the assay was determined by duplicate measurements of drugs. The limit of detection (LOD) for 7 BZs was calculated from a signal-to-noise ratio of 5:1.

Peak RT and area under the curve were checked for standard solution and biological samples using flow rates appropriate for the analytical column and system. All analysis was performed at 30°C. The mean values of RTs and standard deviations were determined.

## RESULTS

Results of analysis were validated statistically and also by recovery studies. The LOD, calculated by using HPLC and UHPLC for 7 BZs, are shown in Table 1.

**TABLE 1:**
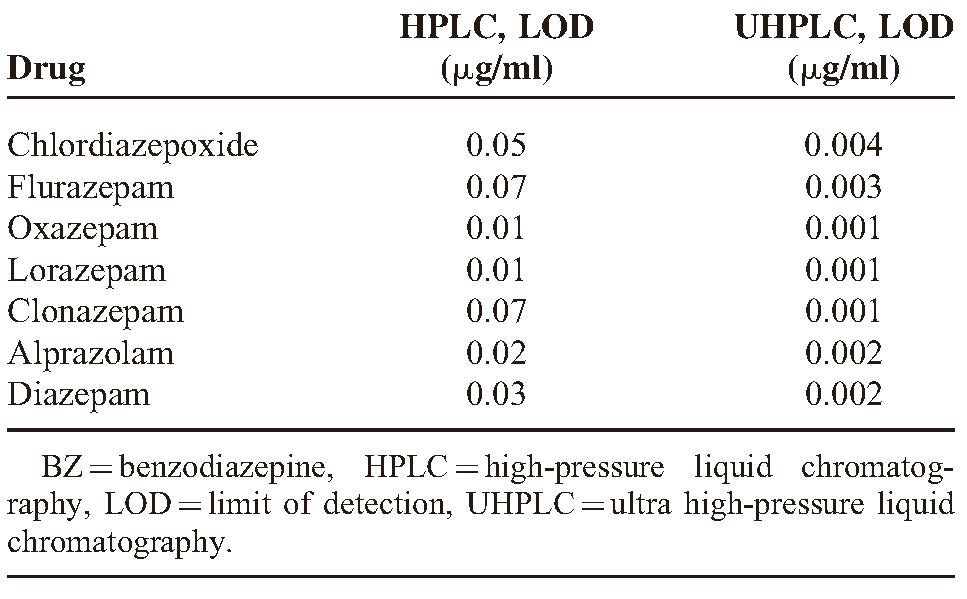
LOD for 7 BZs in HPLC and UHPLC

Linearity for 7 BZs was 0.1 to 10 μg/ml with *R*^2^ > 0.991 in HPLC and 0.01 to 5 μg/ml with *R*^2^ > 0.996 in UHPLC.

The analytical run took 40 minutes with HPLC that is quite a long time for a series of routine analyses in a toxicological laboratory. As chromatographic principles and mechanism of detection in UHPLC and HPLC were the same, method transfer and revalidation were quite easy and especially time saving, as the run time in UHPLC was 15 minutes. The repeatability of analytical results was tested 3 times on each sample and standard solution. RT and standard deviation were calculated on 7 standard solution of BZs (1 μg/ml) and biological samples by HPLC and UHPLC, and their results are shown in Table 2.

**TABLE 2:**
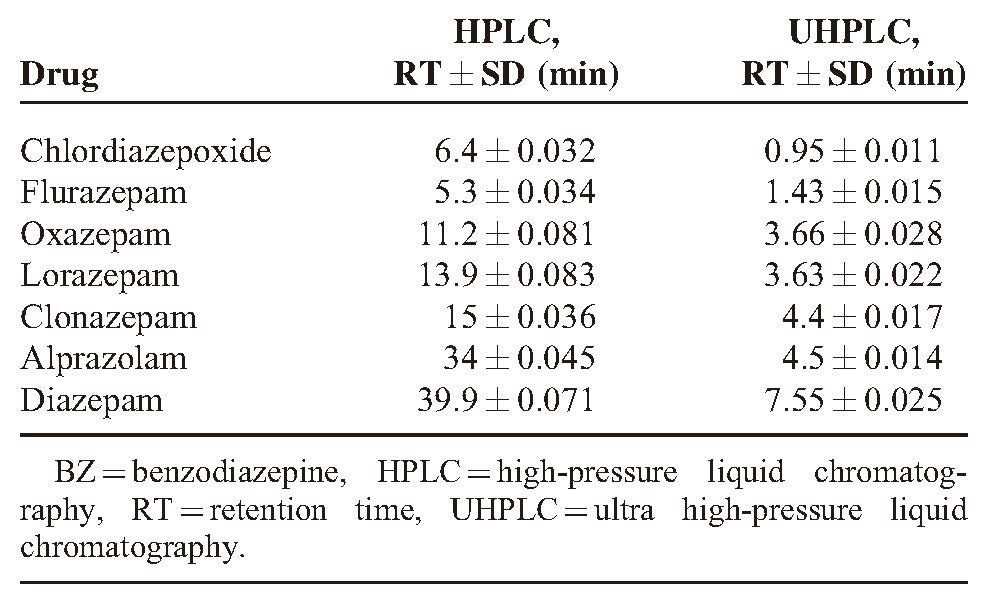
Comparison of RT in Standard Solutions of BZs in HPLC and UHPLC

BZs were detected in 73 biological samples (urine, stomach content, bile, and tissue) in 2012 to 2013. Among 73 samples, diazepam and flurazepam were found in 36 and 18 cases, respectively, and oxazepam was in 2 cases with lower grade of importance.

In one case, pure BZ poisoning was detected as the only cause of death. In 25 cases, multi drug poisoning, such as BZ, TCA, salicylate, methadone, morphine, acetaminophen, and phenothiazine, was the main cause of death, in the remaining 47 cases trauma and other medical disease (such as IHD and CVA) were found as the major factors in causing death and they had received BZ during the treatment course, which was detected in postmortem samples.

Comparison of flow rate, total run time, pressure, volume of mobile phase, volume of sample injection, and particle size of column in HPLC and UHPLC are shown in Table 3.

**TABLE 3:**
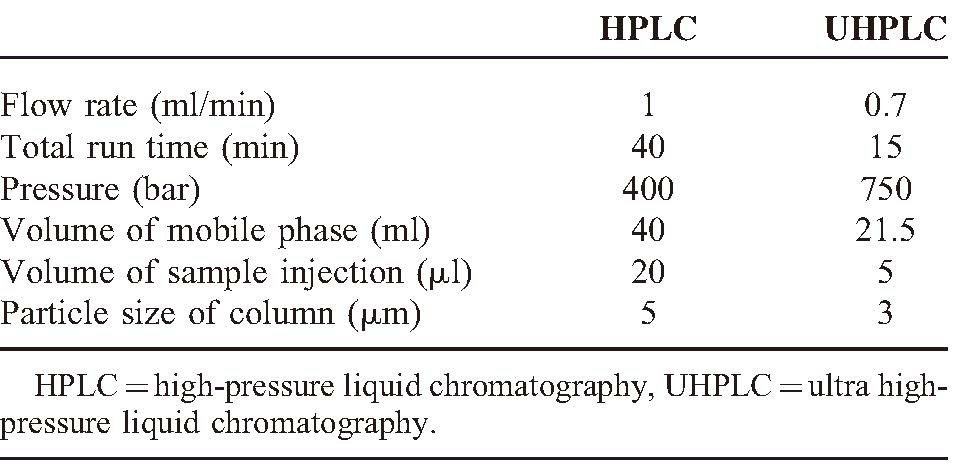
Comparison of Flow Rate, Total Run Time, Pressure, Volume of Mobile Phase, Volume of Sample Injection and Particle Size of Column in HPLC and UHPLC

**FIGURE 2:**
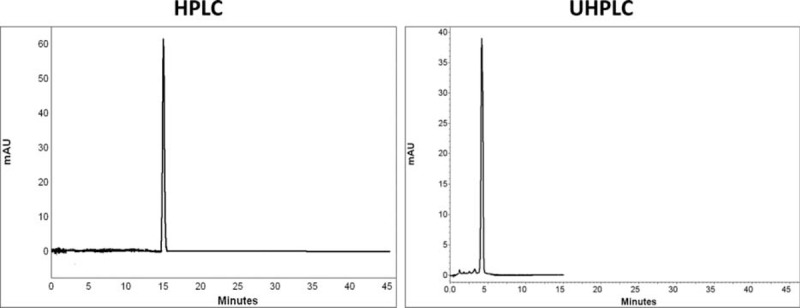
Comparison of chromatograms of clonazepam in HPLC and UHPLC, left and right panels, respectively.

**FIGURE 3:**
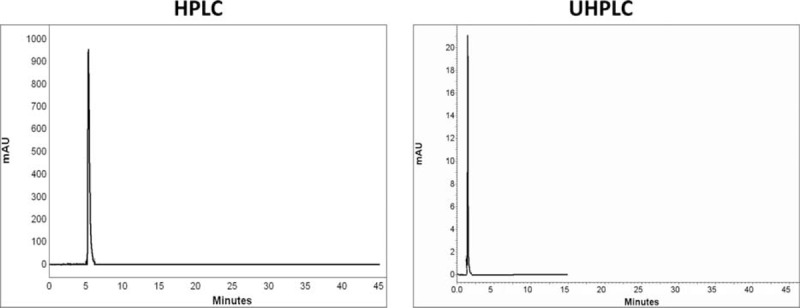
Comparison of chromatograms of flurazepam in HPLC and UHPLC, left and right panels, respectively.

**FIGURE 4:**
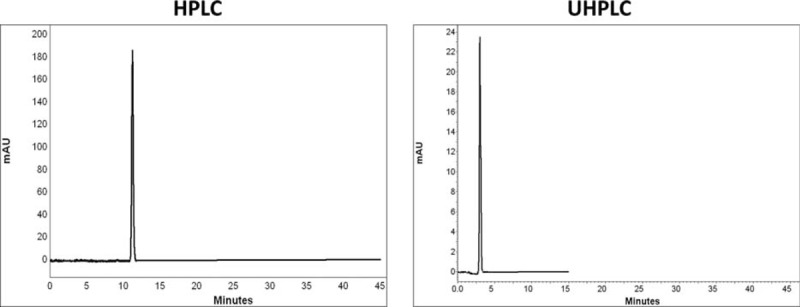
Comparison of chromatograms of oxazepam in HPLC and UHPLC, left and right panels, respectively.

**FIGURE 5:**
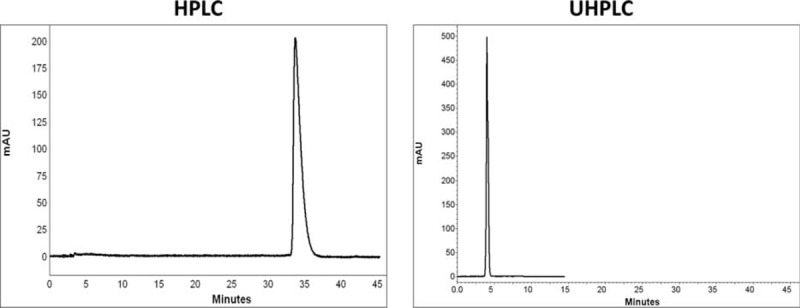
Comparison of chromatograms of alprazolam in HPLC and UHPLC, left and right panels, respectively.

**FIGURE 6:**
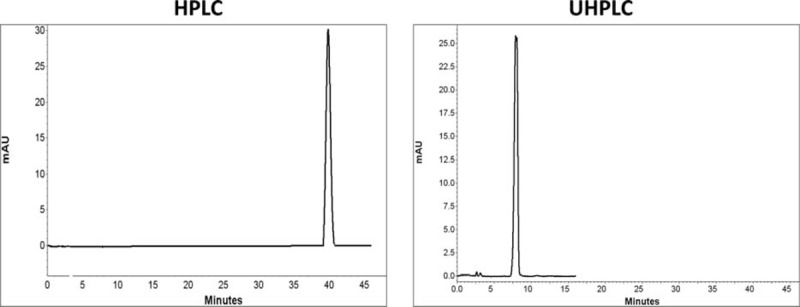
Comparison of chromatograms of diazepam in HPLC and UHPLC, left and right panels, respectively.

**FIGURE 7:**
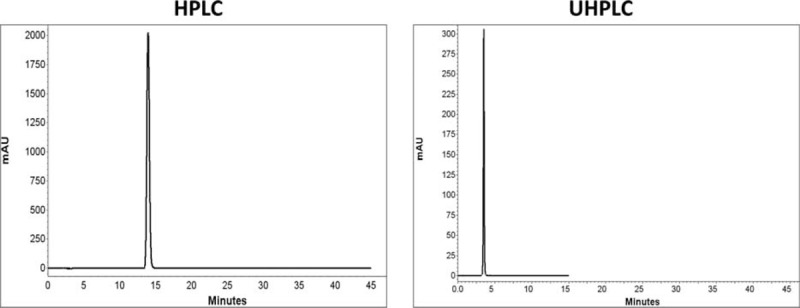
Comparison of chromatograms of lorazepam in HPLC and UHPLC, left and right panels, respectively.

**FIGURE 8:**
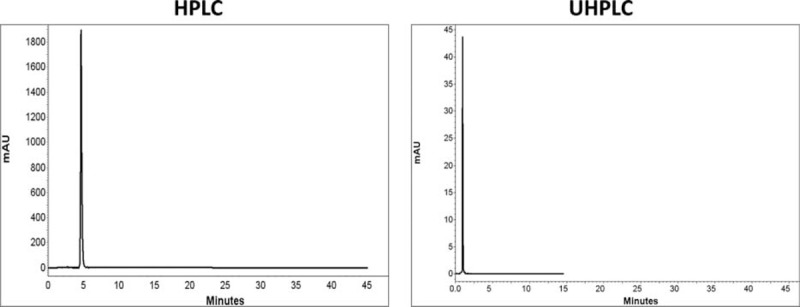
Comparison of chromatograms of chlordiazepoxide in HPLC and UHPLC, left and right panels, respectively.

Comparison of chromatograms of 7 BZs in HPLC and UHPLC is shown in Figures 2 to 8.

## DISCUSSION

BZs need to be monitored in both clinical and forensic laboratories; therefore, an accurate, reliable, and robust method is required to detect and quantify them in biological and nonbiological samples.^1–3^ Nearly, all previously described methods for the analysis of BZs are predominantly intended for toxicological purpose. Mazhar and Binder reported a method for the detection of BZs from serum by using HPLC combined with solid-phase extraction.^9^ Musshoff and Daldrup described an HPLC/PAD method for determining BZ derivatives when used at high doses.^10^ In performing HPLC routine analyses, it is important not only to consider speed, sensitivity, and resolution but also the analysis cost and column maintenance. In an appropriate mobile phase, careful washing of the system and adequate flow rates are needed. Originally, the analytical run took about 40 minutes with a typical back-pressure of about 400 bar. That is quite a long time for a series of routine analyses in a pharmaceutical and forensic laboratory. Therefore, modern developments in liquid chromatography (LC) were applied in order to save time and solvent consumption. As the UHPLC functions according to the chromatographic principles and separation mechanism of HPLC, method transfer and revalidation were quite easy and especially time saving. Since 2004, ultra performance LC (UPLC) has repeatedly demonstrated significant advantages compared with HPLC-based method.^11^

In UHPLC, an ultra high-pressure system allows the use of columns with small particles and small diameter, which has a positive effect on the efficiency and analysis time. Results in our investigation showed that run time in UHPLC is much shorter than routine HPLC (15 min vs. 40 min) and this is in agreement with other studies.^6,11–14^ In our study, the new technique led to reduction in the flow rate of the mobile phase (0.7 ml/min) in UHPLC compared with 1 ml/min in HPLC. As a result of the small column diameter, only low flow rates were applied, but they were sufficient for the effective separation of all compounds in a very short time. Thus, the analysis performed on UHPLC column needed only 21.5 ml of mobile phase, whereas conventional analysis needed 40 ml. Therefore, UHPLC method reduced the flow rate and solvent consumption. Although UHPLC instrument is more expensive than HPLC, due to faster rate of analysis, lower solvent consumption (about half), and shorter run time, cost of analysis in UHPLC is lower than HPLC. UPLC/MS is more sensitive than others but the cost is considerably higher than 2 other techniques. In the routine HPLC method, improving operating speed and efficiency could be gained by reducing the size of the stationary phase packing. In our study, diameter of the packing material in column UHPLC is smaller than HPLC (3 μm vs. 5 μm), which is similar to other investigations.^11,12^ The main difficulty with using smaller diameter packing is that the pressure required to pump the mobile phase through the column increases with the square of particle diameter.^13^ In order to gain the full benefits of small particles, higher operating pressure is required, which can be obtained with standard commercial systems. In UHPLC, 2 pumps provide high pressure, which contributes toward high efficiency and operating speed.

In a Swedish (2003) study, BZs were implicated in 39% of suicides by drug poisoning in the elderly during 1992 to 1996. Nitrazepam and flurazepam accounted for 90% of BZ implicated studies.^15^ In 2010, from a total of 81,427 BZ poisoning in United States, 11 resulted in death.^16^ In many countries, alprazolam was significantly more toxic than other BZs.^17^ In our study, from 73 BZ poisoning, diazepam and flurazepam more frequently caused deaths. Long-acting and rapid effect of diazepam and flurazepam caused increasing consumption and highest fatal toxicity. Oxazepam was found in 2 cases in the presence of diazepam in both of them, but discrimination between oxazepam as a metabolite of diazepam or directly used drug is not possible in routine lab tests. Other metabolites do not use in Iran as a parent drug.

UHPLC method has been used in our laboratory to detect BZs, but quantitative analysis by HPLC and UHPLC and a complete method validation is required.

## CONCLUSION

BZs need to be monitored in clinical and forensic laboratory and detection of them in biological samples is very important. Therefore, we have developed an accurate, sensitive, and fast method for the detection of BZs by using UHPLC. Unlike GC/MS, this technique requires no derivation and due to easy ionization, it is possible to monitor specific fragmentation from the analyses. In comparison to HPLC-based methods, UHPLC has superior chromatographic resolution, enhanced sensitivity, and shorter analytical run times. UHPLC analysis was found to consume less time and less solvent, thus reducing the cost of analysis.

Finally, it can be concluded that the UHPLC analytical system is more convenient and efficient than conventional HPLC in performing complex analyses of BZs.

This research was carried out on Iranian cadavers in Legal Medicine Research Center, LMO of Iran with more than 1.5 million clinical forensic referrals and 50,000 autopsies per year, which is an appropriate field for such researches and trainings.^18–37^
